# Partial Tear of the Common Flexor Tendon and Medial Ulnar Collateral Ligament in a Six-Year-Old After a Minor Traumatic Injury: A Case Report

**DOI:** 10.7759/cureus.102068

**Published:** 2026-01-22

**Authors:** Zainab Almumen, Tareq Almukhlafi

**Affiliations:** 1 Department of Orthopaedic Surgery, Security Forces Hospital Program, Riyadh, SAU

**Keywords:** common flexor origin, elbow injuries, medial ulnar collateral ligament, pediatric elbow, pediatric trauma

## Abstract

Acute traumatic medial ulnar collateral ligament (MUCL) injury, associated with a high-grade sprain of the common flexor tendon and a low-grade sprain of the annular ligament, is an uncommon occurrence in the non-athletic pediatric demographic. At present, there is a lack of high-quality, evidence-based research that details comparable injuries. Furthermore, to our knowledge, there are no established guidelines that provide direction for the optimal treatment of this specific injury.

We report a case involving a six-year-old boy who suffered an acute traumatic MUCL injury to his right elbow. This injury was associated with a high-grade injury to the common flexor tendon, alongside a low-grade sprain of the annular ligament and a minor tear of the ulnar nerve. The patient exhibited persistent pain and a limited range of motion. He received treatment that included a brief period of immobilization, after which he gradually resumed his daily activities without any restrictions. Follow-up visits after one month and three months indicated a complete resolution of pain and a full recovery of the elbow’s range of motion.

## Introduction

Over time, injuries affecting the medial ulnar collateral ligament (MUCL) have become increasingly prevalent. These injuries are particularly frequent among overhead-throwing athletes, owing to repetitive microtrauma caused by valgus stress. The initial documentation of a MUCL injury was by Waris in 1946. The rise in reported cases has enhanced our comprehension of the anatomy and functionality of the MUCL [1].

A thorough understanding of the MUCL’s anatomy and biomechanics reveals that injuries leading to joint instability may result in chronic pain and reduced elbow function, especially in terms of loss of extension, thereby contributing to gradual joint degeneration. In acute or subacute scenarios, however, the most prevalent symptoms include medial elbow swelling alongside tenderness over the ligaments, often accompanied by a complete range of motion. This is noteworthy, given the increased understanding gained over the years and the extensive literature created regarding the management of chronic MUCL injuries in elite overhead athletes. Documented cases of traumatic elbow injuries occurring during acute events are exceedingly rare [2].

Orthopedic surgeons typically adopt a conservative approach in treating many partial and complete MUCL injuries in throwing athletes. A reconstruction procedure developed by Dr. Frank Jobe has been introduced to mitigate complications and enhance the return-to-play rate for professional athletes, where such injuries were once deemed career-ending [3].

In this report, we present an unusual case of traumatic injury to the MUCL of the elbow, which also involved an injury to the common flexor tendon and the annular ligament. To date, there appear to be no known reports in the scientific literature addressing this specific combination of injuries.

## Case presentation

A six-year-old, right-handed male was found to be medically and surgically unremarkable, with no previous incidents of elbow injury. One week later, he visited the pediatric orthopedic outpatient clinic following a fall that impacted his flexed right elbow directly. At the time of the incident, he reported hearing a "pop" sound emanating from his elbow. Subsequently, he experienced intense pain in his right elbow, accompanied by an inability to flex it. When inquired about the delay in seeking medical attention, the parents speculated that it was a simple injury that would likely improve on its own over time. During his initial evaluation at the clinic, the child expressed an inability to flex his elbow beyond 70°. His parents noted a decrease in pain since the initial injury, as reported by both the patient and his family. Standard radiographic imaging of the elbow showed no signs of fracture, significant joint subluxation, or dislocation.

Upon physical examination, mild tenderness was observed in the medial epicondyle region. Active flexion was limited, with the patient achieving 70° and 90° during the passive range-of-motion assessment. Full passive and active extension, as well as pronation and supination, were preserved. During the valgus stress test, approximately 10° of laxity was noted compared to the unaffected side. The distal neurovascular assessment was normal. No treatment was administered; instead, the patient was permitted to continue his daily activities without restrictions, with encouragement to regain a full range of motion in the elbow. A follow-up appointment was scheduled at the clinic for further evaluation.

**Figure 1:**
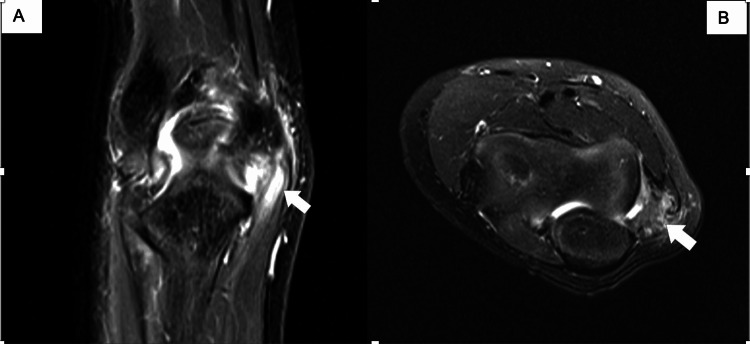
(A, B) MRI of the elbow, showing a high-grade sprain or partial tear involving the common flexor tendon (arrows), on coronal and axial views. MRI, magnetic resonance imaging

**Figure 2:**
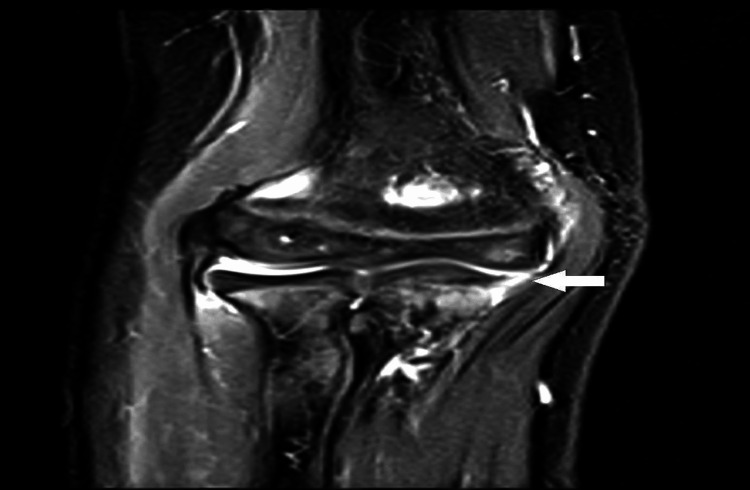
MRI of the elbow showing partial tear of the medial ulnar collateral ligament (arrow). MRI, magnetic resonance imaging

The patient underwent a re-evaluation at the clinic one week following his initial visit. Despite an improvement in symptoms, restricted elbow flexion continued to be evident. Although the patient did not exhibit any signs or symptoms indicative of an infectious etiology, laboratory tests, including C-reactive protein (CRP) and erythrocyte sedimentation rate (ESR), were conducted as part of the infection screening process, with all results falling within normal limits. The patient was scheduled for a plain magnetic resonance imaging (MRI) procedure under general anesthesia the next day. During the examination of the elbow while the patient was anesthetized, restricted flexion was observed, measuring 100°. The MRI findings indicated a high-grade sprain or partial tear of the common flexor tendon and the MUCL (Figures 1-2).

**Figure 3:**
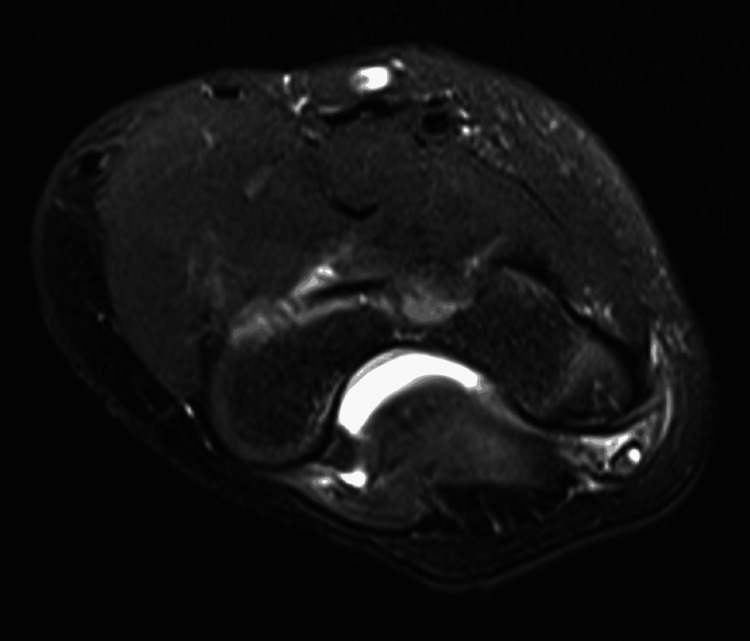
MRI of the elbow showing abnormal signal intensity with a small focal cyst likely presenting as a small tear in the ulnar nerve. MRI, magnetic resonance imaging

Additionally, a low-grade sprain of the annular ligament and abnormal signal intensity involving the ulnar nerve were noted, along with a small focal cyst, likely representing a minor tear (Figure 3).

In light of the MRI findings concerning the ulnar nerve, the plastic surgery team at our institution was consulted for further evaluation. Upon examination, the patient was determined to have a fully intact ulnar nerve; consequently, no additional intervention was deemed necessary or executed regarding the ulnar nerve findings observed in the imaging. In response to the identified ligamentous injury, it was decided to immobilize the elbow using an above-elbow back slab for a duration of two weeks. Following this period, the patient was re-evaluated, and a gradual improvement in the range of motion was noted. The immobilization was lifted, allowing the patient to resume daily activities without restriction. Follow-up visits at one month and three months demonstrated a complete recovery of the elbow’s range of motion.

## Discussion

For the longest time reported in the literature, we succeeded in expanding our knowledge of elbow injuries in an athletic pediatric population with overuse injuries. Although orthopedic surgeons regularly encounter many children with elbow injuries, traumatic ligamentous injury to a skeletally immature elbow poses a challenge, owing to the lack of high-level, evidence-based literature describing such injuries. 

The elbow is a diarthrodial joint consisting of the distal humeral trochlea and capitulum, and it articulates with the proximal ulna and radial head. Joint stability is achieved through the overall joint geometry, active muscle stabilizers, and passive ligamentous stabilizers. When the elbow is flexed to <20° or extended beyond 120°, its congruent articular geometry provides the primary stability. Although articular geometry contributes to stability between 20° and 120°, the MUCL complex plays a significant role in stability against valgus force, acting as the primary passive stabilizer. The dynamic stability of the elbow against valgus force is provided by the flexor carpi ulnaris (FCU), flexor digitorum superficialis, and pronator teres [4].

When studying the pathologies associated with a skeletally immature elbow and its influence on the pattern of injuries, we found that the probability of sustaining medial epicondyle avulsion injuries, compared with MUCL tears, was higher because the physis was weaker than the ligaments [5].

MUCL injuries have been well recognized as a result of chronic, repetitive microtrauma in valgus-producing activities. Acute injury to the MUCL has been described as resulting from a traumatic valgus load on a previously attenuated MUCL. Patients closer to skeletal maturity were more likely to have ligamentous injuries [5-7].

We opted for a trial of conservative management, as supported by both Rettig et al. and Dodson et al., who found that 42% and 90% of patients, respectively, were able to return to their previous functional ability when treated with rest, followed by a gradual return to the throwing program in throwing athletes [6-9]. We reported a paucity of literature describing acute traumatic injuries similar to ours.

Over the years, we have gained a greater understanding of MUCL injuries in throwing athletes; most studies have focused on overhead-throwing athletes, like baseball pitchers. In comparison, when looking into non-throwing athletes with elbow injuries, we found a significant discrepancy in the amount of focus in the literature on those patients, which limits a better understanding of the different mechanisms of trauma sustained to the MUCL in the pediatric population. The mechanism of MUCL injury in non-throwing athletes is mostly the result of acute traumatic incidents. MUCL injuries in throwing athletes are attributed to the repetitive valgus load applied to the elbow, leading to chronic microtrauma, ligament attenuation, and insufficiency. The differences in the nature of the mechanisms and timeline make MUCL injuries in non-throwing athletes a different subset of pathology than the injuries described in throwing athletes, which could indicate a different approach to management. Studies conducted on non-throwing athletes with MUCL injuries, such as professional footballers, gymnasts, and hockey players, who underwent nonoperative treatment based on structured rehabilitation, concluded that players had a higher return-to-play rate than throwing athletes, such as baseball players. In some studies, platelet-rich plasma (PRP) was introduced for MUCL injuries to accelerate healing. The use of PRP injections, adjacent to rehabilitation, to accelerate and facilitate healing was introduced in the literature in an uncontrolled case series [8]. The lack of strong evidence necessitates more controlled studies to prove the benefits of PRP for these injuries. The indications for surgical intervention were limited to those who failed to respond to nonoperative management. Two treatment options are available for MUCL injuries: primary repair and reconstruction.

Although there are limitations in studies comparing primary repair to reconstruction, the available studies demonstrated excellent outcomes and shorter return-to-play periods in patients who underwent primary repair compared to those who underwent reconstruction, with a period of 4-6 months with repair versus 11-20 months with reconstruction [10-12].

## Conclusions

This case report describes the successful, complete recovery of the partial MUCL, accompanied by a high-grade common flexor tendon sprain and a low-grade sprain of the annular ligament, with an asymptomatic small tear of the ulnar nerve. Despite a limitation in mechanical function, this was managed conservatively. Traumatic ligamentous injuries of the elbow joint in nonathletic pediatric populations are not commonly reported. More high-level, evidence-based studies describing similar injuries are required. We emphasize the importance of exploring and documenting such cases, as they can provide valuable insights and bridge the gap in the existing literature on the subject, thus helping surgeons guide patients toward the appropriate management course when facing these injuries.
